# Consensus guidelines for assessing eligibility of pathogenic DNA variants for antisense oligonucleotide treatments

**DOI:** 10.1016/j.ajhg.2025.02.017

**Published:** 2025-03-25

**Authors:** David Cheerie, Margaret M. Meserve, Danique Beijer, Charu Kaiwar, Logan Newton, Ana Lisa Taylor Tavares, Aubrie Soucy Verran, Emma Sherrill, Stefanie Leonard, Stephan J. Sanders, Emily Blake, Nour Elkhateeb, Aastha Gandhi, Nicole S.Y. Liang, Jack T. Morgan, Anna Verwillow, Jan Verheijen, Andrew Giles, Sean Williams, Maya Chopra, Laura Croft, Hormos Salimi Dafsari, Alice E. Davidson, Jennifer Friedman, Anne Gregor, Bushra Haque, Rosan Lechner, Kylie-Ann Montgomery, Mina Ryten, Emil Schober, Gabriele Siegel, Patricia J. Sullivan, Ella F. Whittle, Bianca Zardetto, Timothy W. Yu, Matthis Synofzik, Annemieke Aartsma-Rus, Gregory Costain, Marlen C. Lauffer, the N=1 Collaborative

**Affiliations:** 1 Program in Genetics & Genome Biology, SickKids Research Institute, Toronto, ON M5G 0A4, Canada; 2 Department of Molecular Genetics, University of Toronto, Toronto, ON M5S 1A8, Canada; 3 Division of Genetics and Genomics, Boston Children’s Hospital, Boston, MA 02115, USA; 4 Division of Translational Genomics of Neurodegenerative Diseases, Hertie Institute for Clinical Brain Research and Center of Neurology, University of Tübingen, 72076 Tübingen, Germany; 5 German Center of Neurodegenerative Diseases (DZNE), 72076 Tübingen, Germany; 6 Clinical Molecular Geneticist, Department of Pathology and Laboratory Medicine, Precision Diagnostics Laboratory, Children’s Hospital Colorado, Aurora, CO 80045, USA; 7 Genomics England, London E14 5AB, UK; 8 Department of Clinical Genetics, Cambridge University Hospitals NHS Foundation Trust, Cambridge, Cambridgeshire CB2 0QQ, UK; 9 N=1 Collaborative, 7 Carole Place, Somerville, MA 02143, USA; 10 Institute of Developmental and Regenerative Medicine, Department of Pediatrics, University of Oxford, Oxford OX3 7TY, UK; 11 Department of Psychiatry and Behavioral Sciences, UCSF Weill Institute for Neurosciences, University of California, San Francisco, San Francisco, CA 94158, USA; 12 New York Genome Center, New York, NY 10013, USA; 13 Department of Quantitative Health Sciences, Mayo Clinic, Rochester, MN 55902, USA; 14 Division of Clinical & Metabolic Genetics, Hospital for Sick Children, Toronto, ON M5G 1X8, Canada; 15 Department of Genetic Counselling, Hospital for Sick Children, Toronto, ON M5G 1X8, Canada; 16 Dutch Center for RNA Therapeutics, Department of Human Genetics, Leiden University Medical Center, Leiden 2333 ZA, the Netherlands; 17 Center for Genomic Medicine, Massachusetts General Hospital, 55 Fruit Street, Boston, MA 02114, USA; 18 Department of Quantitative Health Sciences, Mayo Clinic, Rochester, MN 55902, USA; 19 Department of Pathology, University of Utah School of Medicine, Salt Lake City, UT 84112, USA; 20 ARUP Laboratories, Salt Lake City, UT 84108, USA; 21 Ambry Genetics, 1 Enterprise, Aliso Viejo, CA 92656, USA; 22 Rosamund Stone Zander Translational Neuroscience Center, Department of Neurology, Boston Children’s Hospital and Harvard Medical School, Boston, MA 02115, USA; 23 Centre for Genomics and Personalised Health, School of Biomedical Sciences, Faculty of Health, Queensland University of Technology, Brisbane, QLD 4001, Australia; 24 Department of Pediatrics and Center for Rare Diseases, Faculty of Medicine and University Hospital Cologne, University of Cologne, 50937 Cologne, Germany; 25 Max-Planck-Institute for Biology of Ageing and Cologne Excellence Cluster for Ageing-associated Diseases, 50931 Cologne, Germany; 26 Randall Division of Cell and Molecular Biophysics, Muscle Signaling Section, King’s College London, London WC2R 2LS, UK; 27 University College London Institute of Ophthalmology, London EC1V 9EL, UK; 28 UK Platform for Nucleic Acid Therapies (UpNAT), London, UK; 29 Departments of Neurosciences and Pediatrics, University of California, San Diego, San Diego, CA 92093, USA; 30 Division of Neurology, Rady Children’s Hospital, Rady Children’s Institute for Genomic Medicine, San Diego, CA 92123, USA; 31 Department of Human Genetics, Inselspital University Hospital Bern, University of Bern, 3010 Bern, Switzerland; 32 Department for Biomedical Research, University of Bern, 3010 Bern, Switzerland; 33 Department of Clinical Genetics, Erasmus MC, Rotterdam 3015 CN, the Netherlands; 34 Center of Expertise for Neurodevelopmental Disorders (ENCORE), Erasmus MC, Rotterdam 3015 CN, the Netherlands; 35 Great Ormond Institute of Child Health and Queen Square Institute of Neurology, University College London, London WC1N 1EH, UK; 36 Dementia Research Institute, University of Cambridge, Cambridge CB2 0AH, UK; 37 Institute of Medical Genetics, University of Zurich, 8952 Schlieren, Switzerland; 38 Children’s Cancer Institute, Lowy Cancer Research Centre, UNSW Sydney, Sydney, NSW 2033, Australia; 39 Genetics and Genomic Medicine Research and Teaching Department, Great Ormond Street Institute of Child Health, University College London, London WC1N 1EH, UK; 40 Harvard Medical School, Boston, MA 02115, USA; 41 Department of Paediatrics, University of Toronto, Toronto, ON M5G 1X8, Canada

**Keywords:** antisense oligonucleotides, rare genetic diseases, consensus guidelines, individualized genetic therapies, personalized medicine

## Abstract

Of the around 7,000 known rare diseases worldwide, disease-modifying treatments are available for fewer than 5%, leaving millions of individuals without specialized therapeutic strategies. In recent years, antisense oligonucleotides (ASOs) have shown promise as individualized genetic interventions for rare genetic diseases. However, there is currently no consensus on which disease-causing DNA variants are suitable candidates for this type of genetic therapy. The patient identification working group of the N=1 Collaborative (N1C), alongside an international group of volunteer assessors, has developed and piloted consensus guidelines for assessing the eligibility of pathogenic DNA variants for ASO treatments. We herein present the N1C VARIANT (variant assessments toward eligibility for antisense oligonucleotide treatment) guidelines, including the guiding scientific principles and our approach to consensus building. Pathogenic, disease-causing variants can be assessed for the three currently best-established ASO treatment approaches: splice correction, exon skipping, and downregulation of RNA transcripts. A genetic variant is classified as “eligible,” “likely eligible,” “unlikely eligible,” or “not eligible” in relation to the different approaches or as “unable to assess.” We also review key considerations related to assessing the upregulation of transcripts from the wild-type allele, an emerging ASO therapeutic strategy. We provide additional tools and training materials to enable clinicians and researchers to use these guidelines for their eligibility assessments. With this initial edition of our N1C VARIANT guidelines, we provide the rare genetic disease community with guidance on how to identify suitable candidates for variant-specific ASO-based therapies and the possibility of integrating such assessments into routine clinical practice.

## Graphical abstract

**Figure:**
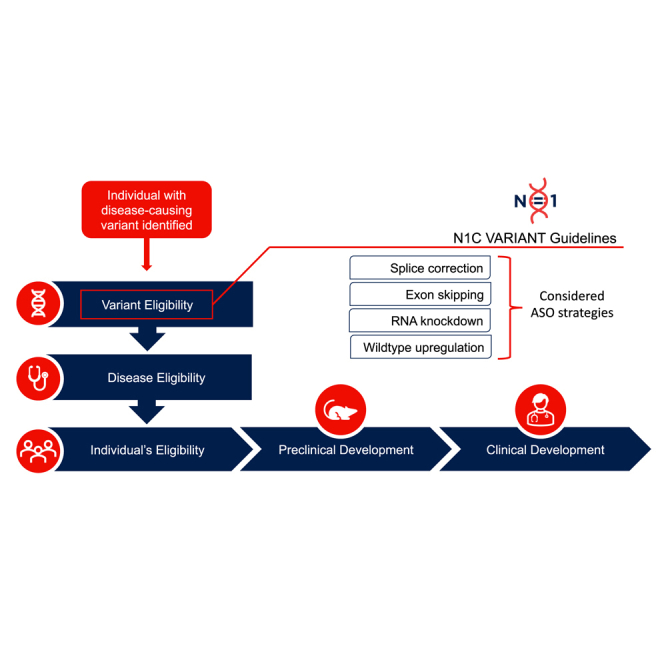

## Teaser

The N=1 Collaborative has developed the N1C VARIANT guidelines, an international consensus guideline for assessing pathogenic genetic DNA variants toward antisense oligonucleotide treatment eligibility. The work includes additional materials such as example assessments and training videos. Further, the N1C provides a variant eligibility calculator to aid with the assessments.

## Introduction

There are around 7,000 different rare diseases known to date, with disease-modifying treatments approved for about 5% of them.^1^^,^^2^ A rare disease is defined as a condition that affects less than 200,000 people in the US or less than 1 in 2,000 individuals within Europe and Canada.^1^ It is estimated that 6% of the world’s population lives with a rare disease.^3^ The majority of rare diseases are thought to be genetic in origin, and with the massive improvements made in genetic diagnostics in the last decades, we can now diagnose up to 50% of individuals who suffer from a rare disease.^4^ As more individuals receive a molecular genetic diagnosis, the need to develop targeted treatments is increasingly urgent. However, because many of these rare diseases only affect a handful of individuals across the globe, the usual drug development route is not a viable pathway in most cases, and more bespoke therapeutic strategies are necessary.^5^

Antisense oligonucleotides (ASOs) are one promising form of genetic therapy. Over 20 different oligonucleotide therapies for general applications have been approved by the US Food and Drug Administration (FDA), the European Medicines Agency (EMA), the UK’s Medicines and Healthcare Products Regulatory Agency (MHRA), and/or the Japanese Ministry of Health, Labour and Welfare.^6^ Additionally, these drugs have been administered and well studied in thousands of people worldwide. Systemic delivery is possible (for instance via subcutaneous or intravenous injection), but localized or targeted delivery is also feasible for a growing number of target organs (brain and spinal cord via intrathecal injection, eye via intraocular injection, and liver and muscle via GalNAc and transferrin receptor targeting, respectively), allowing relatively low doses to be administered with potentially high treatment effects.^7^ Because of the finite half-life of ASOs, treatment needs to be administered repeatedly (often every 1–4 months), but this also allows the treatment regimen and dosing to be tailored to each individual where helpful, optimizing individual benefits.

ASOs are versatile in their usage, as they can be employed to (1) downregulate transcripts in the case of toxic gain-of-function (GoF) and dominant-negative variants, (2) restore the reading frame in the case of truncating variants leading to a loss-of-function (LoF) effect, (3) correct aberrant splicing, and (4) increase protein expression of the wild-type (WT) allele in disorders associated with haploinsufficiency (HI; see Data S1).^8^^,^^9^^,^^10^ Hence, ASOs can be used to target specific genetic variants present in groups of individuals, with group sizes being as small as  n = 1. However, not all genetic variants can be targeted with ASOs; even the ones that can be targeted can be distinguished into more eligible (stronger) and less eligible (weaker) candidates. Thus, it is important to systematically assess every pathogenic DNA variant for its eligibility for ASO treatment to identify the individuals most likely to benefit from such therapies.

Since 2018, multiple groups and organizations have developed customized ASO treatments for individuals targeted to their specific variant, a single-nucleotide polymorphism, or the disease gene in general.^11^^,^^12^^,^^13^ These developments have given hope that individualized, disease-modifying therapies might be a realistic option in the near future for others in the rare-disease community. As of January 2025, to our knowledge, 27 individuals have received individualized ASO therapies, and more are under development .

The N=1 Collaborative (N1C) (https://www.n1collaborative.org/) is a global initiative to develop best practices for ultra-rare “*n* = 1/few” therapy development and promote safe and equitable access for individuals with rare diseases. The N1C patient identification working group (PIWG) is one of several workgroups organized by the N1C. The PIWG focuses on three key areas: (1) identifying suitable genetic variants for ASO development, (2) determining diseases that are prime candidates for genetic therapy, and (3) identifying individuals who are suitable for individualized genetic therapy development.

Individualized ASO therapy development can be split into three distinct parts (see graphical abstract), with the assessment of the individual at the beginning of the development process. This assessment is based upon three main pillars: (1) assessment of the genetic variant for molecular eligibility, (2) assessment of the disease, and (3) assessment of the individual (disease stage, symptoms, and goals). We have discussed the different aspects of this evaluation process extensively elsewhere.^8^^,^^14^^,^^15^^,^^16^^,^^17^^,^^18^

To aid with the prioritization of individuals for ASO developments, the PIWG has developed criteria and established a consensus on assessing diagnostic DNA variants for amenability to ASO therapies. The guidelines are intended for clinical geneticists and clinicians working with rare-disease individuals, diagnostic laboratories, researchers, and research institutes working on rare genetic disorders and aim to help them identify and prioritize amenable disease-causing variants so they can assess individuals for further ASO development.^14^

Here, we describe the development of the consensus guidelines—named the N1C VARIANT (*v*ariant *a*ssessments towa*r*d elig*i*bility for *an*tisense oligonucleotide *t*reatment) guidelines—present the first version of the guidelines, and provide training materials such as example assessments and training videos. We further introduce the “N1C variant eligibility calculator,” which aids with variant evaluations.

## Guideline and resource development

### Overview of guideline development

The development of the consensus guidelines (N1C VARIANT guidelines) was a multisite effort that took input from researchers and genetics healthcare providers. The guidelines were developed through alternating rounds of revision and piloting, leading to the final version 1.0 (Data S1).

#### Version 0.1

Development began with an internal assessment of sample variants by the PIWG. A single assessor from four participating sites (the Dutch Center for RNA Therapeutics [DCRT], Leiden, the Netherlands; the Hospital for Sick Children [SickKids], Toronto, Canada; the Hertie Institute for Clinical Brain Research, Tübingen, Germany; and Boston Children’s Hospital [BCH], Boston, USA) independently assessed 30 selected variants (previously assessed at the DCRT). The assessment approaches and outcomes from each site were compared, debated by the PIWG, and distilled into an outline of the guidelines.

This outline proposed the purpose, content, format, and definitions of classifications. The outline was shared with the PIWG membership for input and revised based on their feedback. This outline was then used to draft the first version of the consensus guidelines (version 0.1). This version was only applicable to LoF variants in genes causing autosomal recessive and X-linked recessive disorders and only assessed variants for exon skipping and splice-correcting ASOs. This draft was shared with the PIWG, and feedback was collected and applied. After revisions by the PIWG, the revised draft was shared with a group of external volunteers (*n* = 5) who reviewed the guidelines, provided feedback, and assessed a test set of three variants (Table S1).

#### Version 0.2

Feedback and assessment results from the external volunteers were collected as written responses and used for further revision (version 0.2). We paid attention to not only the feedback on the guidelines but also how the test variants were assessed and whether the reasoning for the assessments was in alignment with our guidelines. When assessors did not come to the correct conclusion, we rephrased and adjusted the guidelines to better aid with the assessments.

Version 0.2 was once again shared with the PIWG for edits and feedback before being distributed to a larger group of external volunteer assessors (*n* = 14) for a second round of piloting on a set of 12 test variants (Table S1). Once again, feedback and assessment reports were collected and used for revising the guidelines.

#### Version 0.3

In version 0.3, the guidelines were expanded to include assessments of eligibility for ASO or small interfering RNA (siRNA)-mediated transcript knockdown of GoF and dominant-negative variants and for upregulation from the WT allele (e.g., targeted augmentation of nuclear gene output [TANGO]^19^). Additionally, the work was expanded to include assessments of other inheritance patterns (excluding mitochondrial inheritance). The revised guidelines were shared with external volunteers (*n* = 19) for a third round of piloting with 15 test variants (Table S1). Assessment results and feedback were collected as written responses and used by the PIWG to refine the guidelines. This final version (version 1.0) was shared with all co-authors for final feedback before submission.

### Test variant curation

All test variants were selected by one member of the PIWG who did not participate in the assessment rounds. Selected variants were sourced from published literature or the ClinVar database (https://www.ncbi.nlm.nih.gov/clinvar/). Two PIWG members independently assessed each variant and determined the feasibility of the variant assessment depending on the publicly available information (including prior success with ASO development, where applicable). Once a variant’s analysis and classification were agreed upon, these members drafted a “correct answer” representing the expected outcome using the guidelines (Data S2). Answers with explanations were communicated to all volunteer assessors after each assessment round.

### Guideline piloting

Volunteer assessors ranged from graduate students (at both the master’s and PhD levels) to faculty with different levels of experience in clinical genetics and ASO therapy development. Assessors included trained basic science or translational researchers, genetic counselors, and clinicians. Assessors were recruited via the professional networks connected to the N1C (e.g., departmental colleagues, announcements in the N1C newsletter, and international conferences featuring N1C PIWG members). An overview of the assessors’ roles and affiliations is given in Table 1.

**Table 1:** Global makeup of volunteer assessors, including current institution and role or position

**Country of institution**	**No. of assessors**	**Institutions**	**Role(s)/position(s)**
Australia	2	Children’s Cancer Institute	PhD candidate
		Queensland University of Technology	senior scientist
Canada	4	The Hospital for Sick Children	masters research students (3), genetic counselor
Germany	3	Hertie Institute for Clinical Brain Research	postdoctoral research fellow
		University of Cologne	clinician scientist, MD candidate
The Netherlands	2	Dutch Center for RNA Therapeutics, LUMC	PhD candidate
		Erasmus Medical Center	MD/PhD candidate
Switzerland	2	University Hospital of Bern	senior scientist
		University of Zurich	senior scientist
United Kingdom	4	University College London	associate professor, professor of neuroscience and clinical geneticist, research associate
		Genomics England	clinical fellow
United States	8	Boston Children’s Hospital	genetic counselor, clinician scientist
		Mayo Clinic	research fellow, senior bioinformatician
		Ambry Genetics	genetic counselor
		Massachusetts General Hospital	genetic counselor
		Rady Children’s Hospital	clinician scientist
		Children’s Hospital Colorado	clinical molecular geneticist

### Video example development

To support assessors, exemplary assessments of selected test set variants were provided via short videos. The video examples provide step-by-step instructions on assessing variants. The videos were designed and recorded on Microsoft PowerPoint. The videos were reviewed by both the PIWG and the volunteer assessors. A subset of videos was first shared with the PIWG, who provided feedback on the content and structure. The videos were then revised before being shared with volunteer assessors during each round of piloting. Feedback on the videos was collected as written responses.

The variants discussed in the video examples were selected by members of the PIWG. All selected variants were sourced from published literature or the ClinVar database, with some already having a developed ASO. Two members of the PIWG compared analyses and determined the feasibility of the variant assessment approach. Once a variant’s analysis and classification were agreed upon, a step-by-step analysis was recorded, along with the expected classification of the variant. Videos are available on the N1C YouTube channel (https://www.youtube.com/playlist?list=PL1FIwS0tbJHj0-aDMmZ5fUy5d40eiwa8B) and N1C website (https://www.n1collaborative.org/post/n1c-variant-guidelines).

### Development of the N1C variant eligibility calculator

An interactive decision tree was developed to facilitate applying the guidelines (the N1C variant eligibility calculator). First, a catalog with questions and answers based on the guidelines was written, including indications of connections between different sections. For the development of an interactive online tool, HTML and Javascript code was written based on the catalog of questions with the help of ChatGPT, which provided a skeleton of the code upon request. The tool was deployed on the N1C website (http://eligibilitycalculator.n1collaborative.org/). The full code is available on the N1C’s GitHub page: https://github.com/N1Collaborative/Variant-Eligibility-Calculator. The eligibility calculator was thoroughly tested by several co-authors of this manuscript, including multiple assessors doing their assessments of the final 15 variants using the tool to see if they came up with the correct answers. Feedback was gathered through a written response and incorporated accordingly.

### Upregulation from the WT allele table

The N1C VARIANT guidelines refer to various resources for the assessment of pathogenic variants toward upregulation from the WT allele (Data S1).^19^^,^^20^^,^^21^^,^^22^ To aid in the assessment of variants toward upregulation eligibility, a combined file containing the findings from each suggested paper was generated (Table S2). The data from Mittal et al., Lim et al., and Felker et al. were extracted from the papers’ supplemental files.^19^^,^^20^^,^^21^ The data from Liu et al.^22^ were extracted from the uORF website (http://rnainformatics.org.cn/RiboUORF/) through POST requests. All data from all available genes from each paper were combined into one Excel file. For each gene in the combined file, pLI scores and ClinGen HI scores were indicated. The pLI score was downloaded from gnomAD v.4.0 (https://gnomad.broadinstitute.org/downloads). ClinGen HI scores were downloaded from ClinGen (https://search.clinicalgenome.org/kb/downloads). Additionally, genes in the combined file were annotated with the names of their corresponding antisense transcripts using the HGNC database (https://www.genenames.org/). Antisense long non-coding RNAs were identified based on the presence of the terms “antisense” or “regulatory RNA” in the “name,” “alias_name,” or “prev_name” fields, and annotated matches were included in the “HUGO antisense” column.

## Outcomes

### Purpose of guidelines

We have developed consensus guidelines (N1C VARIANT guidelines) for eligibility assessment and prioritization of (likely) pathogenic DNA variants for ASO treatments. With these guidelines, assessors can identify genetic variants most likely to benefit from an ASO-based therapy and distinguish these variants from currently less suitable candidates. The full guidelines are available in Data S1. Updated versions of the guidelines will also be available via the N1C website (https://www.n1collaborative.org/post/n1c-variant-guidelines).

The guidelines take into consideration the genetic diagnosis of the individual, molecular principles, and pathomechanism of the disease and genetic variant. The purpose of the guidelines is to provide professionals working in the rare genetic disease field, e.g., clinicians, diagnostic laboratories, and rare disease researchers, with a framework for analyzing and classifying disease-causing variants for their amenability to ASO-based therapy. With these guidelines, assessors should be able to do the following.

(1) Identify pathogenic variants eligible for assessment and use publicly available databases and resources to assist in the variant assessment process.
(2) Assess whether a pathogenic variant is eligible for ASO-mediated splice correction (i.e., correction of mis-splicing).
(3) Assess whether a pathogenic variant is eligible for ASO-mediated exon skipping.
(4) Assess whether a candidate gene and/or variant is eligible for siRNA- or ASO-mediated transcript knockdown.
(5) Classify variants as “eligible,” “likely eligible,” “unlikely eligible,” or “not eligible” for the aforementioned ASO approaches or as “unable to assess” using these guidelines. The definition of each classification is dependent on the type of ASO therapy.
(6) Consider strategies for the upregulation of WT alleles in cases of HI.

Overall, these guidelines focus on evaluating (likely) pathogenic, disease-causing variants for eligibility for ASO treatment, thus addressing the first step in the evaluation of an individual for ASO therapy development. For a full assessment beyond the variant, disease- and individual-specific clinical factors have to also be taken into consideration,^14^^,^^15^^,^^16^^,^^17^^,^^18^ which is beyond the scope of these guidelines. These guidelines provide a detailed and practical explanation of how to assess DNA variants. Besides the general guidance, the guidelines list and indicate relevant exceptions where applicable.

### Guideline structure

Only a subset of the guidelines will be relevant to evaluating any one specific variant. The guidelines walk readers through a series of steps where they are prompted to verify variant annotations, inheritance patterns, and pathomechanisms. If critical information is unavailable or insufficient, then the reader is prompted that this variant is ineligible for further assessment (unable to assess). Conversely, if the necessary information is available and known, then readers can use the guidelines to identify appropriate or multiple applicable ASO strategies: splice correction, canonical exon skipping, RNA knockdown, or upregulation from the WT allele. Upon identification of a relevant ASO strategy, readers can direct themselves to the relevant section with the help of flowcharts, where they can further assess the variant’s eligibility toward a specific strategy in greater detail. Throughout the guidelines, relevant, publicly available resources to aid with the assessments are shared.

Furthermore, assessors are encouraged to check whether an ASO has already been developed for a specific variant, exon, or disorder (whether in clinical or preclinical stages). Clear criteria are provided to define what constitutes sufficient evidence for a functional ASO, depending on the strategy. To assist in this search, resources and recommended search terms are offered.

At the end of the assessment, assessors can classify a variant’s eligibility toward splice correction, canonical exon skipping, and/or RNA knockdown based on the information gathered. The classification criteria were developed as part of the consenting process and are outlined below. Assessment strategies are provided for upregulation from the WT allele, but classifications toward eligibility are not defined because this area of ASO therapeutics is less established to date. Instead, the guidelines provide context on when upregulation from the WT allele might be used and considerations for their development. We further provide a resource to check for multiple WT upregulation approaches at once by combining available resources from the literature into one simple Excel table (Table S2).

### Classification terms

The complexity of the assessments necessitated defining terms for a classification schema. Prior published classification schema assessed variant amenability to ASO therapy using terms such as “probably,” “possibly,” or “unlikely” amenable; “exclude from assessment”; and “consider for exon skipping.”^12^^,^^18^ However, these schemas focused on only certain types of variants and ASO strategies. To improve on these prior schemas and expand them to multiple ASO strategies, version 1.0 of these guidelines now employs five tiers for all classifications: eligible, likely eligible, unlikely eligible, not eligible, and unable to assess.

Eligible variants are those for which functional evidence supports the effectiveness of an ASO approach. For splice-correcting ASOs, this means that an ASO has already been developed and shown to be effective, either clinically or preclinically, for the specific splice-altering variant. In the context of exon skipping, which aims to “skip” the exon containing the pathogenic variant to produce a truncated yet functional protein product, one would search for functional evidence that the exon skipping was non-pathogenic. This would include experimentally induced exon-skipping events (i.e., CRISPR deletions and ASOs) showing functional evidence at the protein level that residual protein function remains. This also includes naturally occurring exon skipping events (i.e., benign exon-skipping events or exon deletions found in healthy individuals). A pathogenic variant found within an exon where either experimentally induced or naturally occurring exon skipping occurs would then be classified as eligible for canonical exon-skipping ASOs. Similar ideas apply when assessing variants for eligibility toward knockdown. If a GoF or dominant-negative variant, both of which can be considered for knockdown, is found on a gene where a knockdown approach has been functionally proven, the variant can be classified as eligible for knockdown ASOs.

Not eligible variants are those for which a specific ASO therapeutic approach is not considered possible. This may be due to functional evidence demonstrating the failure of ASO therapies (for example, canonical exon skipping led to a non-functional protein) or molecular criteria that render the variant unsuitable for ASO targeting. Examples of not eligible variants include single-exon genes in the context of exon-skipping ASOs or genes with tightly regulated dosage in the context of RNA knockdown strategies.

Likely eligible variants are those that, based on molecular criteria, could potentially be targeted by an ASO, although no functional evidence is currently available to confirm this. Conversely, unlikely eligible variants are those where molecular criteria suggest an ASO is unlikely to be effective but no functional evidence directly contradicts the potential use of an ASO.

Variants are classified as unable to assess when they either do not apply to these guidelines (e.g., are of a type that cannot be assessed) or if there is not enough information available that allows for an assessment of the variant (e.g., the inheritance pattern of the variant is unknown or no information on the pathomechanism is available).

### Training videos

At the time of publication, 12 training videos have been created and shared (Table 2). These videos provide step-by-step guidance for assessors, highlighting key resources and assessment techniques. Each video demonstrates the assessment of specific variants toward a relevant strategy, with each example leading to a unique outcome. The training videos can be accessed via the N1C YouTube channel: https://www.youtube.com/playlist?list=PL1FIwS0tbJHj0-aDMmZ5fUy5d40eiwa8B.

**Table 2:** List of video examples referred to and published with version 1.0 of the N1C VARIANT guidelines

**Video**	**Variant**	**Gene symbol**	**OMIM**	**ASO strategy**	**Outcome/classification**
1	c.2626C>T (GenBank: NM_000350.3; p.Gln876^∗^)	*ABCA4*	601691	canonical exon skipping	eligible
2	c.597-1340A>G (GenBank: NM_016589.4; p.?)	*TIMMDC1*	615534	splice correcting	eligible
3	c.680dup (GenBank: NM_000533.5; p.Cys228Leufs^∗^5)	*PLP1*	300401	canonical exon skipping	not eligible
4	c.213+1G>C (GenBank: NM_003793.4; p.?)	*CTSF*	603539	splice correcting	not eligible
5	c.611A>G (GenBank: NM_000277.3; p.Tyr204Cys)	*PAH*	612349	splice correcting	unlikely eligible
6	c.815-27T>C (GenBank: NM_025152.3; p.?)	*NUBPL*	613621	splice correcting	unlikely eligible
7	c.3503_3504del ( GenBank: NM_024312.5; p.Leu1168Glnfs^∗^5)	*GNPTAB*	607840	canonical exon skipping	unlikely eligible
8	c.5645G>A (GenBank: NM_001040142.2; p.Arg1882Gln)	*SCN2A*	182390	knockdown	eligible
9	c.3733C>T (GenBank: NM_001165963.4; p.Arg1245^∗^) and c.67C>T (GenBank: NM_130839.5; p.Arg23^∗^)	*SCN1A* and *UBE3A*	182389 and 601623	upregulation from the WT allele	eligible
10	c.264del (GenBank: NM_003793.4; p.Cys89Alafs^∗^59)	*CTSF*	603539	canonical exon skipping	likely eligible
11	c.538C>T (GenBank: NM_000170.3; p.Gln180^∗^)	*GLDC*	238300	canonical exon skipping	not eligible
12	m.13084A>T (GenBank: NC_012920.1), c.748A>T (Gencode: ENST00000361567.2; p.Ser250Cys) and c.597-1340T>G (GenBank: NM_016589.4; p.?), and c.1120T>C (GenBank: NM_001040142.2; p.Phe374Leu)	*MT-ND5 and TIMMDC1* and *SCN2A*	516005615534 and 182390	N/A	unable to assess

Variants normalized using Mutalyzer (https://mutalyzer.nl/) and VariantValidator (https://variantvalidator.org/). Video examples can be accessed via the N1C YouTube channel: https://www.youtube.com/playlist?list=PL1FIwS0tbJHj0-aDMmZ5fUy5d40eiwa8B.

### Variant eligibility calculator

To support the assessment and help assessors focus on going through the sections of the guidelines relevant to their current assessment, we developed the N1C variant eligibility calculator. At the time of publication, the eligibility calculator walks assessors step by step through version 1.0 of the N1C VARIANT guidelines. One key feature of the calculator is the inability to skip question prompts. Each step discussed in the guidelines is crucial for in-depth variant assessment and is required for accurate variant classification. Due to the inability to progress without answering the question, users of the calculator are encouraged to further research the gene or variant before proceeding with the assessment. Additionally, the calculator takes into consideration the variant type (i.e., missense, stop gain, or synonymous) before directing users to relevant sections of the guidelines. Overall, this tool allows users to systematically navigate the guidelines, acting as a “checklist” before proceeding with assessments. The calculator further provides users with the ability to track their assessments and receive a printout of the specific questions and corresponding answers to understand the overall classification and identify potential mistakes during the assessment process.

### Guideline maintenance

To ensure the guidelines remain up to date and reflect the current state of ASO technologies, the PIWG will conduct a yearly review.

During this review, the guidelines will be revised to implement new tools/databases/websites where available and to adjust if new knowledge relevant to any part of the guidelines has been made available. The updated version will then be reviewed by the PIWG members and subsequently released to the community. Simultaneously, all training materials, videos, and the eligibility calculator will be updated to reflect the latest version of the guidelines. The version number and date of the latest update will be indicated on the guidelines and calculator.

In the case of scientific breakthroughs that warrant immediate changes in the assessment process, the PIWG will agree to an unscheduled update outside of the yearly cycle.

The PIWG is continually expanding, with diverse stakeholders and experts actively contributing to ongoing work in the rare-disease space. The scheduled revisions ensure that analyses remain timely and reflect the most current insights of the field.

## Discussion

Here, we introduced version 1.0 of the N1C VARIANT guidelines for the assessment of (likely) pathogenic DNA variants for eligibility toward ASO treatments, alongside the process of their development and consenting. Additionally, we introduce and discuss the development of training materials and tools to aid with variant assessments.

These guidelines represent an international consensus approach for evaluating the potential eligibility of pathogenic DNA variants causing monogenic disorders toward ASO therapies. With the significant progress and publicity in the last few years regarding the development of individualized genetic therapies,^11^^,^^12^^,^^13^ there is hope within the rare-disease community that ASOs may benefit an increasing number of community members. We aim to support the rare-disease community by providing guidance on which variants are most likely to be eligible for ASO therapies.

As a takeaway from the iterative process of developing these guidelines, we want to emphasize that it takes time to become familiar with the assessment procedure. Similar to the annotation and classification of pathogenicity for genetic variants with the ACMG guidelines,^23^ the assessment of pathogenic variants for their eligibility for ASO treatments takes many different aspects into account and requires practice. With the help of our volunteer assessors, we have ensured that all steps required for assessment are clearly outlined and can be followed by different professionals without prior experience in variant assessment for ASO development.

We propose a five-tier classification schema for ASO treatment amenability of DNA variants, whereby a variant is classified with respect to specific ASO strategies and can thus receive different labels for different types of ASO treatments. The classifications are eligible, likely eligible, unlikely eligible, not eligible, or unable to assess. Although the eligible and not eligible definitions are clear, categorizing and classifying variants as likely eligible and unlikely eligible proves to be more challenging because the evidence for or against eligibility exists on a spectrum. Future work will aim to refine these categories further, mirroring the efforts to introduce more gradations into the ACMG/AMP variant classification scheme.^23^

With the planned yearly updates, we expect to communicate adjustments in the upcoming years. Especially as technologies advance and knowledge grows, we expect that some variant classifications for certain ASO approaches will change with these developments. This could be, for example, that new information on the feasibility of ASO designs for certain types of variants becomes available, e.g., the recently shown allele-selective gapmer ASO targeting *KIF1A* (MIM: 601255).^13^ This will necessitate the reassessment of some variants over time. In that regard, we would like to point out that it is important to read the literature on available ASO treatments critically and scrutinize the methodology used and the functional data provided before considering a variant eligible for ASO treatments.

At this point, a limited number of ASOs are developed and clinically tested; thus, ASOs are not currently available for all different variant types, pathomechanisms, and inheritance patterns discussed herein. That means that no gold-standard evidence for many considerations was available for the establishment of these guidelines. Thus, this work is based on the collective expertise gained from assessing about 1,500 variants since 2018 at the sites of the PIWG members (BCH, SickKids, and DCRT) and the knowledge the PIWG members have on human molecular genetics and ASO design and development, which involves individuals with extensive expertise in the field of ASO development and tailored ASO therapies.

Although efforts were made to test the guidelines with various assessors from different professional backgrounds, their application in diverse healthcare settings might reveal additional needs for adjustments and refinements in the future, and any such refinements will be incorporated with the scheduled updates.

Although the amenability of a specific variant to a certain ASO therapeutic strategy is a fundamental first step, disease- and individual-specific factors are equally important considerations in the development and provision of individualized genetic therapies.^8^ Considerations will also include the reversibility and severity of symptoms. This means that an eligible variant does not necessarily equal an eligible person. The assessments of disease- and individual-specific factors are outside the scope of this work and will require additional recommendations.^24^^,^^25^

Ideally, we would like to see the integration of the eligibility assessments into clinical practice to provide individuals suffering from rare genetic diseases not only with a diagnosis but also with information on possible treatment approaches, where applicable. We believe that with the additional training material and test variants provided, clinical geneticists and specialist human geneticists working in laboratories and diagnostic centers can train themselves to become assessors. In the long term, we envision automation of our guidelines and assessment procedures in the form of tools that take the pathogenic variant as input and deliver an analysis of the best therapeutic strategy for each individual.

## Data and code availability

Data are available in Tables S1 and S2 and Data S2 for variant assessments and upregulation from the WT allele approaches. Original data for Table S2 are available in Lim et al., Mittal et al., Felker et al., and Liu et al.,^19^^,^^20^^,^^21^^,^^22^ as described in the guideline and resource development section.

The code generated during this work is available on GitHub under an open-source AGPL license (https://github.com/N1Collaborative/Variant-Eligibility-Calculator).

## Acknowledgments

We would like to thank additional N1C members for their input and help during the development of these guidelines. We especially want to thank Nicole Nolen for all her help and support in making the training material and tools available on the N1C website. We also thank all families and clinicians who over the years have provided us with their genetic diagnoses, which ultimately led to the establishment of these guidelines. This work was supported by the 10.13039/501100000780European Union, project European Rare Disease Research Alliance (ERDERA, #101156595) (to M.S. and A.A.-R.). D.C. is supported by a SickKids Restracomp Master’s Scholarship, the SickKids Innovators Fund, and SickKids Precision Child Health. L.N. is supported by a Canada Graduate Scholarship - Master’s from the Canadian Institutes of Health Research (CIHR). D.C., B.H., and G.C. also acknowledge support from CIHR (PJT186240). D.B. is supported by a Humboldt Research Fellowship and the Hertie Network of Excellence in Clinical Neuroscience. M.C.L. is funded by a Walter Benjamin Fellowship (DFG, #521414448). A.E.D., K.-A.M., E.F.W., and M.R. are supported by the UK Platform for Nucleic Acid Therapies (UpNAT,Medical Research Council MR/Y008405/1). B.Z., A.A.-R., and R.L. are supported by a ZonMW PSIDER grant. S.J.S. is supported by the HDR-UK Molecules to Health Records Driver Programme.

## Author contributions

D.C. and M.C.L. of the N1C PIWG conceptualized the N1C VARIANT guidelines. D.C. and M.C.L. drafted the guidelines and the manuscript, conducted the assessment rounds, and evaluated and implemented the feedback. D.C. made the example videos. M.C.L. selected the test variants for assessment and developed and built the variant eligibility calculator. M.M., D.B., C.K., L.N., A.L.T.T., A.S., E.S., S.J.S., M.S., T.Y., A.A.-R., G.C. are members of the N1C PIWG; they reviewed the outlines and drafts of the different rounds of guidelines and edited the manuscript. They further provided feedback on the videos. M.M., D.B., C.K., and L.N., also participated in the variant assessment training and provided feedback and explanations on the assessments. S.L. facilitated the PIWG meetings, set up documents, and helped with putting the work onto the public databases and the N1C website. E.B., N.E., A.G., N.S.Y.L., J.T.M., A.V., J.V., A.G., S.W., M.C., L.C., H.S.D., A.E.D., J.F., A.G., R.L., K.-A.M., M.R., E.S., G.S., P.J.S. participated as assessors and provided feedback on the guidelines and training material. B.H. made the upregulation from the WT table. L.N., E.F.W., and B.Z. tested the variant eligibility calculator and provided feedback. The authors are listed in the author line as follows: junior members of the N1C PIWG were named first, followed by assessors who participated in multiple rounds of assessments and provided extensive feedback; all other assessors were named in alphabetical order. Lastly, senior members of the N1C PIWG were named. D.C. and M.C.L. of the N1C PIWG were placed as first and senior authors, respectively, as they were leading the overall effort.

## Declaration of interests

The authors declare no competing interests.

## Web Resources

GenBank, https://www.ncbi.nlm.nih.gov/genbank/
Gencode, https://www.gencodegenes.org
GitHub, https://n1collaborative.github.io/Variant-Eligibility-Calculator/
N1C – eligibility calculator, http://eligibilitycalculator.n1collaborative.org/
N1C – variant guidelines, https://www.n1collaborative.org/post/n1c-variant-guidelines
OMIM, http://www.omim.org
YouTube, https://www.youtube.com/playlist?list=PL1FIwS0tbJHj0-aDMmZ5fUy5d40eiwa8B

## Supplemental information

Data S1. The N1C VARIANT guidelines

Data S2. Answer key to all test variants

Table S1. Test variants

Table S2. Upregulation from the WT allele summary table

Document S2. Article plus supplemental information
