# Supplementary material for: Consensus guidelines for assessing eligibility of pathogenic DNA variants for antisense oligonucleotide treatments
Source: Am J Hum Genet. 2025 Mar 25;112(5):975–83. doi: 10.1016/j.ajhg.2025.02.017 (PMC12120168; doi:10.1016/j.ajhg.2025.02.017)
Supplement: Table S1. Test variants [file mmc3.pdf]

Table S1

Table with test variants for piloting rounds. The table indicates which test variants were provided to the assessors for the 3 piloting rounds. This purposely includes variants with incorrect descriptions.

Piloting Round	Transcript	Gene	Variant
**1**	NM_025152.3	*NUBPL*	c.815-27T>C
**1**	NM_000350.3	*ABCA4*	c.769-784C>T
**1***	NM_024312.5	*GNPTAB*	c.3505_3504del
**2**	NM_020366.3	*RPGRIP1*	c.1468-128T>G
**2**	NM_000329.3	*RPE65*	c.1430A>G
**2**	NM_001086521.2	*NDUFAF8*	c.195+271C>T
**2**	NM_003907.3	*EIF2B5*	c.1156+13G>A
**2**	NM_206933.4	*USH2A*	c.2692C>T
**2**	NM_000391.4	*TPP1*	c.225A>G
**2**	NM_024298.5	*MBOAT7*	c.758_778del
**2**	NM_018075.5	*ANO10*	c.289del
**2***	NM_024312.5	*GNPTAB*	c.3503_3504del
**2**	NM_003650.4	*CST7*	c.2035-946G>A
**2**	NM_000303.3	*PMM2*	c.640-15479C>T
**2**	NM_000202.8	*IDS*	c.1122C>T
**3**	NM_018075.5	*ANO10*	c.1025G>A
**3**	NM_001127222.2	*CACNA1A*	c.4174G>A
**3**	NM_024312.5	*GNPTAB*	c.3488del
**3**	NM_133433.4	*NIPBL*	c.5329-15A>G
**3**	ENST00000361390.2	*MT-ND1*	m.4142G>T
**3**	NM_014727.3	*KMT2B*	c.8079delC
**3**	NM_024312.5	*GNPTAB*	c.1123C>T
**3**	NM_005859.5	*PURA*	c.159dup
**3**	NM_001167623.2	*CACNA1C*	c.1216G>A
**3**	NM_000561.4	*HEXB*	c.1509-26G>A
**3**	ENST00000435607.3	*SCN4A*	c.3891C>A
**3**	NM_001244008.2	*KIF1A*	c.914C>T
**3**	NM_000492.4	*CFTR*	c.2989-313A>T
**3**	NM_001194.4	*HCN2*	c.736G>A
**3**	NM_000170.3	*GLDC*	c.538C>T

*Indicates duplicate variants assessed in multiple rounds
